# Publishing interdisciplinary research – a perspective from the *Croatian Medical Journal*

**DOI:** 10.3325/cmj.2015.56.399

**Published:** 2015-10

**Authors:** Srećko Gajović

**Affiliations:** 1 University of Zagreb School of Medicine, Croatian Institute for Brain Research, Zagreb, Croatia; 2 *Croatian Medical Journal*, Zagreb, Croatia

Like Archimedes who demanded a place to stand so that he could move the Earth, scientific journals also need a firm point to stand on. We use this example because it stresses the importance of determining the journal’s specific research field by editorial policies. One of the basic strategies for medical journals is to define their scope, which helps them to target specific readership, attract authors, and select reliable reviewers. Simply put, it governs editorial policy. The *Croatian Medical Journal* is a general medical journal and although it might seem at first that lacking a specific field is mainly a weakness (1), we will try to provide arguments that it does not necessarily have to be so.

The dilemma whether to be general or specific is not only to be considered by editorial policies, but also by the researchers. Aspiring for grant agencies support, and favorable publication metrics, research groups are equally concerned whether to be specific or general, in particular if they pursue interdisciplinary research. Biomedical research is particularly resource demanding, since it requires sophisticated equipment, consumables, and highly skilled experts. To obtain and maintain these resources scientists are under constant pressure to acquire and justify investments. Alongside high-end publications, successful teams pursue other values, including translational skills connecting basic and clinical medicine, technology transfer skills documented by patents, and societal impact. The impact of the research for the benefit of the society is a category that is still difficult to be objectified and evaluated (2). However, it is continuously gaining importance in the current research environment. The demand to evaluate the wider impact of science is seen as a threat to curiosity-oriented research, and blue skies research, but it is inevitable if we are to preserve and increase funding needed for advancement of biomedical research (3). Curiosity-driven research is the basic substance of science and should therefore be incorporated into overall research activities, regardless of whether they are mandate-driven or applied research. This impact-driven change can be perceived as an opportunity rather than a threat, giving science an energizing role in the knowledge-based society. As a consequence, the research is expected to be regulated in a way that the obtained novelty makes a change for the benefit of society.

The request for a research project to achieve an impact is not at all simple and results in increasing complexity of research and in projects combining more research fields. Responding to this request transforms the research environment. In biomedicine it is an uphill battle to acquire increasing amounts of resources and to obtain results that provide benefits for the patients. This pressure has a double effect; on the individual level, it requires researchers who are excellent in their own field, but on the research group level there is a need to team up and gather diverse competencies in a coordinated effort. These diverse competencies drive research increasingly toward a manifold disciplinary effort. It can be multidisciplinary, interdisciplinary, or transdisciplinary corresponding to additive, interactive, or holistic approach respectively (4). Regardless of the level and type of collaboration, the need to combine disciplines in order to achieve the desired result is constantly present.

Although reasons for multiple disciplinary efforts are well justified, on the practical level the interdisciplinary research confronts difficulties, among others lack of support from both granting agencies as well as scientific journals (5). In particular, to choose the right journal for multidisciplinary publications can be a challenge, requiring from the authors to pinpoint the center of gravity of their research, and then approach the correct journal. The author of this editorial himself received an unfavorable peer-review of a multidisciplinary publication, in which the reviewer considered only a single segment of the research and failed to grasp the main message that was outside of his or her own particular expertise. To put it simply, the real value of multidisciplinary research can be difficult to evaluate, not to mention that such research may find it hard to get financial support. The excellence of a reviewer in his own field can undermine fair judgment of a publication or a grant proposal when it comes to the research components outside that reviewer’s field of interest.

From the editorial perspective, the *Croatian Medical Journal* welcomes multiple disciplinary efforts, and recognizes this as an important asset to its future. As a journal oriented to emerging scientific communities and researchers, it represents an exemplary forum to present results not to be assigned to a particular field. As it is claimed here, the future of scientific research lies in the combination and coordination of multiple competences by uniting highly skilled scientists and specialists (6). Taking this in account the emerging research teams can choose to shortcut their development starting immediately as a multidisciplinary effort. Instead of first gaining recognition in separate fields and then aspiring to the cross-disciplinary effort, they can use the platform offered by the *Croatian Medical Journal* to present their coordinated efforts from the very beginning. The already established quality tradition of the *Croatian Medical Journal* can represent an advantage over the newly established journals, corresponding to the emerging cross-disciplinary fields (eg, personalized medicine, regenerative medicine, tissue engineering) (7). One of the examples is nanomedicine, a lucrative field with a number of emerging journals, which aspire indexing and rapidly achieve high impact. To compete with these emerging journals is a challenge that stands before general medical journals like the *Croatian Medical Journal*.

If multiple disciplinary effort is the right place to stand, then the *Croatian Medical Journal* can aspire “to move the Earth” of biomedicine. At a new interdisciplinary stage of the biomedical future, the *Croatian Medical Journal* must be ready to respond to the emerging challenges of the new research pattern and publish relevant science.
